# Response to: Emergency Medicine Residents Experience Acute Stress While Working in the Emergency Department

**DOI:** 10.5811/westjem.2021.2.52121

**Published:** 2021-04-28

**Authors:** Adam J. Janicki, Stephanie O. Frisch, P. Daniel Patterson, Aaron Brown, Adam Frisch

**Affiliations:** * University of Pittsburgh, School of Medicine, Department of Emergency Medicine, Pittsburgh, Pennsylvania; † University of Pittsburgh, School of Nursing, Department of Acute and Tertiary Care, Pittsburgh, Pennsylvania

We first want to thank Peters and colleagues for their interest in our work. They bring up two notable points in discussing our study.

We utilized three-lead Nasiff CardioHolter monitors to assess physiologic parameters. The raw data was downloaded directly from Holter monitors using Nasiff software and then reviewed by study authors to ensure quality data was obtained. While there was some motion artifact throughout the shift, the majority of the data was reliable with discernible QRS complexes in one of the three leads. We did not quantify the amount of artifact in each reading. This data was then analyzed using the provided software. When designing the study, we felt that a three-lead Holter, although less convenient and comfortable, would afford us additional data over pulse rate sensors. We do acknowledge as a limitation that we cannot account for all data obscured by artifact as we cannot control how the software decides to analyze and provide specific summary measures.

While prior literature has shown heart rate (HR) and heart rate variability (HRV) change during acute stress, their use as a proxy for stress is imperfect with no true gold standard.^1^ This is evidenced by how different studies operationalize various measures of HRV using different time points. Physical activity while on shift was normally limited to mild intensity walking, standing, and sitting, as evidenced by our HR data (mean 78 beats per minute, maximum 114 beats per minute during clinical work), but we acknowledge that even mild intensity activity may have affected HR and HRV. Prior work has demonstrated that any exercise will decrease HRV and increase HR, but there appears to be an intensity dose-response with HRV reaching a minimum at moderate-high intensity – sustained heart rates of 120–160 beats per minute for 3–10 minutes.^2^ Obtaining additional baseline data during mild exertion would certainly clarify our findings, but we also acknowledge that there are numerous variables that affect HR and HRV such as time of day, exertion, hydration status, life stressors, age, gender, etc, and controlling for all of these was not feasible.

Future studies should focus on disentangling the physical activity component of HRV versus the effects of acute stress via interaction with patients in an emergency setting to confirm our findings. We hope that our qualitative data helps inform future work evaluating the acute stress response and the impact of acute stress on EM resident performance.
